# Early soft tissue response to zirconium oxide and titanium healing abutments in vivo: a study in dogs

**DOI:** 10.1186/s12903-021-01748-0

**Published:** 2021-08-24

**Authors:** Min Wang, Shuang Zhang, Longjie Chen, Haixiao Zou, Yining Wang, Haibin Xia

**Affiliations:** 1 The State Key Laboratory Breeding Base of Basic Science of Stomatology (Hubei-MOST) & Key Laboratory of Oral Biomedicine Ministry of Education and Department of Oral Implantology, School & Hospital of Stomatology, Wuhan University, Wuhan, 430079 China; 2 The State Key Laboratory Breeding Base of Basic Science of Stomatology (Hubei-MOST) and Key Laboratory of Oral Biomedicine Ministry of Education and Department of Preventive Dentistry, School and Hospital of Stomatology, Wuhan University, Wuhan, 430079 China; 3 Lanzhou Hospital of Stomatology, Lanzhou, 730000 China; 4 Department of Stomatology, Second Affiliated Hospital of Nanchang University, Nanchang University, Nanchang, 330006 China; 5 The State Key Laboratory Breeding Base of Basic Science of Stomatology (Hubei-MOST) and Key Laboratory of Oral Biomedicine Ministry of Education and Department of Prosthodontics, School and Hospital of Stomatology, Wuhan University, Wuhan, 430079 China

**Keywords:** Peri-implant crevicular fluid, Healing abutment, Ligation, Zirconium oxide, Titanium

## Abstract

**Background:**

This study aimed to investigate the clinical characteristics and early soft tissue response to zirconium oxide (Zr) and titanium (Ti) healing abutments in dogs.

**Methods:**

Eight implants (four at each hemi-mandible) were inserted after bilateral mandibular third and fourth premolars and first molar extraction in dogs. Then, two Zr and two Ti healing abutments were connected to each unilateral mandible eight weeks later. The ligation method was used to create a peri-implant mucositis model and the 24 abutments were divided into four groups: Zr or Ti healing abutments with ligation (ZrL, TiL) or non-ligation (ZrN, TiN). The clinical indices, peri-implant crevicular fluid (PICF), and inflammatory cytokines (TNF-α and IL-1β) were measured and analyzed on days 0 and 28. The dogs were then sacrificed on day 28, soft tissues around the implants were harvested, and inflammation infiltration was tested by immunohistochemistry. Normal distribution test and two-way analysis of variance was used to analyze the data.

**Results:**

The results showed that the clinical indices were similar for Zr and Ti healing abutments. There was significantly more PICF in the ZrL and TiL groups compared to in the ZrN and TiN groups. The TNF-α levels in PICF were significantly different between ZrL and ZrN groups on day 28. And the TNF-α levels in PICF were significantly higher in TiL group on day 28 than that on day 0. However, the number of inflammatory cells was not significantly different between the groups as measured by immunohistochemistry.

**Conclusions:**

These data indicate that soft tissue responses to Zr healing abutments with peri-implant mucositis were comparable to those of Ti healing abutments in vivo, providing a theoretical foundation for the clinical application of Zr abutments.

## Background

Soft tissues serve as a protective barrier between the oral environment and the underlying peri-implant bone, and proper integration of soft tissues significantly affects the long-term success of implant-supported restorations [1–3]. Various hazards, including bacterial accumulation, overloading, and prosthetic manipulation, adversely affect the attachment of peri-implant soft tissues to abutments [2, 4, 5]. Furthermore, the biocompatibility of the transmucosal part of implants is crucial for ensuring a high quality of attachment between the mucosa and abutment.

Over the past few decades, titanium (Ti) has become the gold standard material for dental implants and implant abutments due to its excellent biocompatibility, mechanical strength, and corrosion resistance in complex oral environments [6–8]. However, its potential defects have also attracted the attention of dentistry. On the one hand, Ti abutments can hardly meet patients’ increasing esthetic requirements for implant-borne restorations, thus biomaterials with better optical properties are greatly needed. On the other hand, Ti can release sub-micrometer particles into the oral cavity, which can induce inflammatory cytokine secretion in vivo [9], or even cause potential hypersensitivity towards Ti in a limited number of patients [10]. Therefore, increasing esthetic demands have driven the fabrication of tooth-colored ceramic implant abutments [11, 12]. Recently, yttrium oxide-stabilized zirconium oxide (Zr) has gained increasing attention for its excellent esthetic properties, mechanical properties, and ideal biocompatibility [13, 14]. Importantly, fewer bacterial colonies have been reported on Zr surfaces than on Ti surfaces in vitro studies [15–17]. Furthermore, less soft tissue inflammation infiltration has been reported in response to Zr healing caps compared to Ti healing caps [18].

Nevertheless, an ideal implant abutment material should have the ability to maintain long-term homeostasis of the peri-implant mucosal microenvironment, there are several studies on the responses of peri-implant soft tissue to abutments [1, 18–21]. However, most of these studies were conducted without inflammatory challenges. There are few studies on the responses of soft tissue to abutments in inflammatory environment. Therefore, the purpose of this study was to investigate the clinical characteristics and early soft tissue response to Zr and Ti healing abutments with or without peri-implant mucositis induced by ligation. This study fills the current gap in Zr abutment research (clinical evaluation in an inflammatory environment) and provides a solid theoretical foundation for its clinical application.

## Methods

The study protocol was approved by the Ethics and Institutional Animal Care and Use Committees of the School and Hospital of Stomatology, Wuhan University. This study conformed to the Arrived guidelines.

### Animals

This study was performed on three 1-year-old female beagle dogs weighing 12.5–15 kg. The dogs were purchased from Hubei Anlu Dog Farm (Hubei, China) and were individually housed and maintained on a commercial diet and water ad libitum. Their health was checked and maintained daily.

### Implant design and surfaces

A total of 24 implants with a cylinder design, 8.0-mm length, and 3.5-mm diameter were fabricated from grade 2 unalloyed Ti rods. An inner threaded hole was made to fit the Zr and Ti healing abutments, and the implants were then ultrasonically washed first in acetone, then in ethanol, and finally in deionized water; this process was repeated three times. The surfaces were sandblasted and acid-etched as previously described [22].

### Surgery procedures

**Fig. 1:**
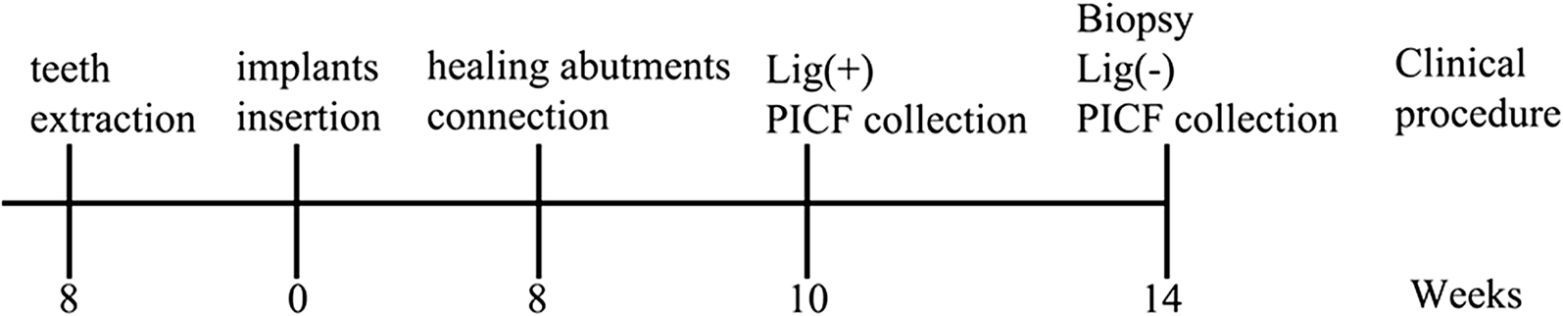
Outline of the experiment

The experimental schedule is shown in Fig. 1. All surgical procedures were performed under general anesthesia using intravenous sodium pentobarbital (3%, 1 mL/kg; Merck, Germany). Local instillation with 1–2-ml Primacaine adrenaline (Acteon, France) was administered for hemostasis and to reduce postoperative pain. Streptomycin and penicillin were administered postoperatively for four days.

**Fig. 2:**
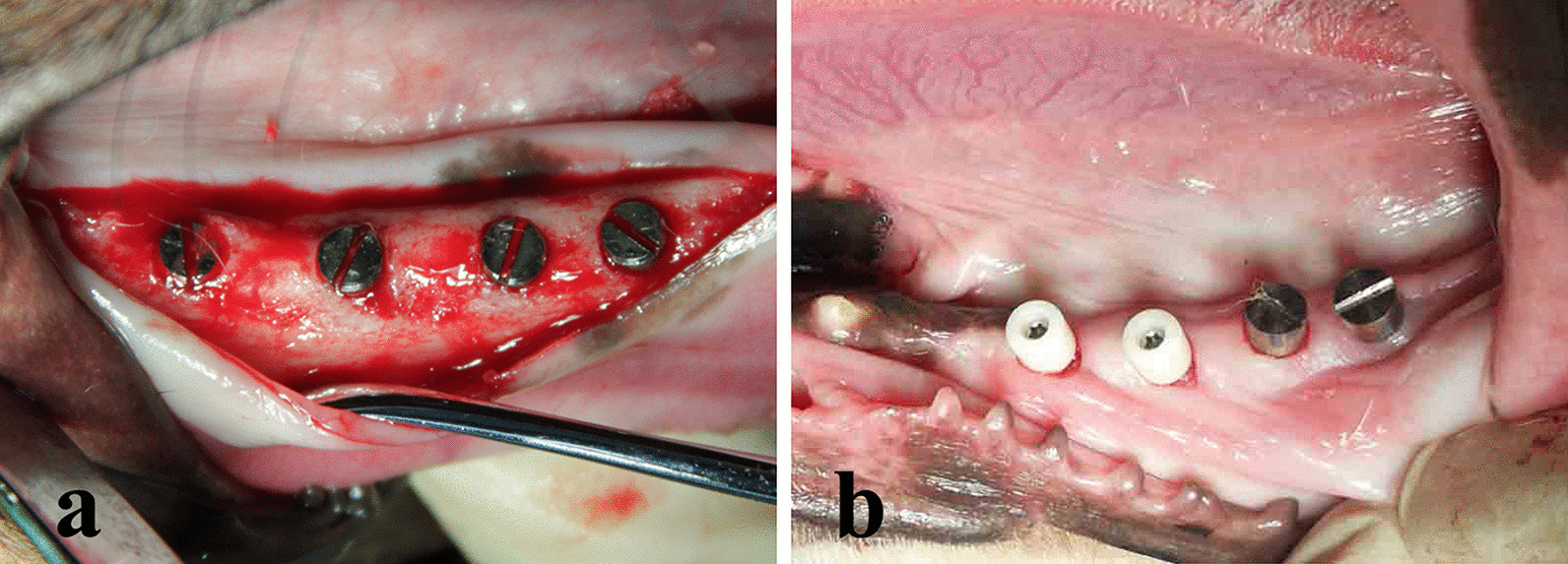
**a** Occlusal view of the implant insertion in the unilateral mandibular edentulous region. **b** Occlusal view of the Zr and Ti healing abutments connection

After two weeks of adaptive feeding, the mandibular third and fourth premolars and first molar (P3-M1) were bilaterally extracted. After eight weeks [23], a full-thickness mucoperiosteal flap was evaluated, and eight implants, four at each hemi-mandible, were inserted in each dog. In total, 24 implants were used across the three dogs. The implants were placed with their coronal margins at the level of the alveolar bone crest (Fig. 2a). Cover screws were installed, and the flaps were sutured.

After another eight weeks of healing, the implants were exposed using a circular scalpel. Then, two Zr and two Ti healing abutments were connected randomly to each unilateral mandible (Fig. 2b) and oral hygiene maintenance was initiated. After two weeks, the plaque index (PI), gingival index (GI), and probing depth (PD) were recorded, and PICF was collected as baseline (day 0). Then, silk threads were placed at the neck of the healing abutments randomly on one side for each dog to promote plaque accumulation as the ligation groups [24, 25]. On the contralateral side, the healing abutments were carefully cleaned using Colgate dentilave every two days as the control groups. The implants were divided into four groups: Zr healing abutments with ligation (ZrL) (n = 6), Ti healing abutments with ligation (TiL) (n = 6), Zr healing abutments without ligation (ZrN) (n = 6), and Ti healing abutments without ligation (TiN) (n = 6). Then 28 days later, the PI, GI, and PD were recorded, and PICF was collected.

### Clinical measurements

Clinical measurements were obtained at six sites around the healing abutments on days 0 and 28. The PI [26] and GI [27] were initially scored, followed by PICF sampling, and finally, PD was recorded. All clinical examinations were performed by one examiner.

### PICF sampling and processing

Any supragingival plaque attached to healing abutments was gently cleaned using wet cotton balls. The implants were then isolated using cotton rolls and gently air-dried, and PICF was collected using 8 × 2-mm filter paper strips (Whatman #3, United States). The paper strips were inserted into the mesial- and distal-buccal sulcus of the healing abutment until slight resistance was felt and then remained there for 30 s. Any strips contaminated with bleeding or exudates were discarded. The volume of PICF was calculated by weighing and subtracting the value before and after PICF collection using a precise electronic balance. The strips were then stored at − 70 °C until further analysis.

### Enzyme-linked immunosorbent assay (ELISA) analysis

The PICF samples were thawed and eluted according to the Griffiths’ method [28]. Two ELISA kits (CATA00, DY3747, R&D, USA) were used to determine the levels of TNF-α and IL-1β. All procedures were performed according to the manufacturer’s instructions. Absorbance at 450 nm was measured using an ELISA reader (BioTeK Instruments, Inc., Winooski, VT, USA). The levels of TNF-α and IL-1β were estimated using the standard assay criteria. All tests were performed in duplicates.

### Histopathological analysis

The dogs were sacrificed by administering a lethal overdose of pentobarbital sodium. The hemi-mandibles were removed and fixed in 10% buffered paraformaldehyde (pH 7.2) for 48 h. The specimens were carefully dissected into pieces and decalcified in a 10% ethylenediaminetetraacetic acid (EDTA) solution at 4 °C until a syringe needle could punch through encountering no resistance. The implants were carefully removed, and all specimens were embedded in paraffin blocks. Specimens were sectioned along their longitudinal axis at 5 µm and stained with hematoxylin and eosin (HE) for histological examination. Then, the slices were observed under a conventional light microscope (Olympus BHS-313, Tokyo, Japan) at ×20 magnification.

To identify the early inflammatory infiltration of soft tissues around the Zr and Ti healing abutments, immunohistochemistry using the avidin–biotin–peroxidase method was performed on 5-μm-thick sections. After deparaffinization and rehydration, the sections were subjected to antigen retrieval using citrate buffer (10 mM, pH 6.0, using a pressure cooker for 5 min at 120 °C) and endogenous peroxidase was blocked using 3% H_2_O_2_ for 10 min at 37 °C. Slides were preincubated with a protein block solution (2% skim milk, 0.05% Triton X-100, and phosphate-buffered saline [PBS]) for 30 min at room temperature to prevent nonspecific binding. Immunostaining was performed by incubating the slides with primary monoclonal antibodies against TNF-α (1:50; R&D, USA) and IL-1β (1:100; NOVUS, USA) in a humid chamber at 4 °C overnight. The reactions were developed using diaminobenzidine, and the immunostained sections were counterstained with hematoxylin. For the control experiments, the primary antibodies were replaced with PBS. Sections were observed under a conventional microscope (Olympus BHS-313, Tokyo, Japan) and photographed using a calibrated digital camera (Olympus C-35AD-4, Tokyo, Japan) at ×20 and ×40 magnification. The different markers were quantified using a specific image analysis software (Photoshop CC2019; Adobe System, San Jose, CA, USA).

### Statistical analysis

Data are presented as mean ± standard deviation (SD). Differences between the Zr and Ti implant healing abutments, with or without ligation, were evaluated using normal distribution test and two-way analysis of variance. Statistical significance was set at *p* < 0.05. All statistical analyses were performed using SPSS (version 19.0; SPSS, Inc., Chicago, IL, USA).

## Results

### Clinical findings

**Fig. 3:**
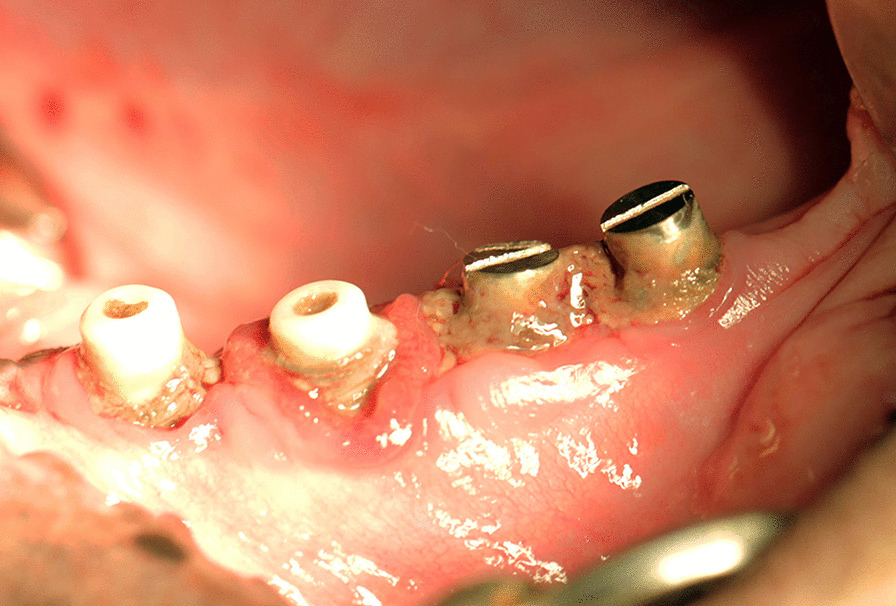
The occurrence of peri-implant mucositis at day 28

Visual assessment confirmed the presence of peri-implant mucositis (Fig. 3). Soft tissues around the Zr and Ti healing abutments were red and inflamed at the fourth week after ligation. No differences were observed in the PI and PD among the four groups on days 0 and 28 or in the GI of tissues around the Zr and Ti healing abutments on day 0. However, the GI of tissues around the ZrL and TiL healing abutments was significantly higher than that around the ZrN and TiN abutments on day 28 (Table 1).

**Table 1:** Clinical parameters of Zr or Ti healing abutments with or without ligation

		Day 0			Day 28		
		L	N	*P*	L	N	*P*
PI	Zr	1.2 ± 0.98	1.8 ± 1.17	0.282	2.3 ± 0.52	2.5 ± 0.55	0.534
	Ti	2.2 ± 0.98	2.3 ± 1.03	0.785	2.7 ± 0.52	3.0 ± 0.00	0.220
	P	0.113	0.417		0.220	0.072	
GI	Zr	0.3 ± 0.5	1.0 ± 0.6	0.051	1.5 ± 0.6	0.5 ± 0.5	0.002*
	Ti	0.3 ± 0.5	0.5 ± 0.5	0.609	1.5 ± 0.6	0.8 ± 0.4	0.011*
	P	1.000	0.135		1.000	0.431	
PD (mm)	Zr	3.13 ± 0.43	2.92 ± 0.38	0.622	3.45 ± 0.64	3.08 ± 0.66	0.108
	Ti	2.75 ± 0.69	3.58 ± 1.20	0.068	3.75 ± 0.96	3.3 ± 0.63	0.644
	P	0.386	0.139		0.490	0.617	

Normal distribution test and two-way analysis of variance were applied

(*PI* plaque index, *GI* gingival index, *PD* probing depth, *L* ligation, *N* non-ligation, *P* P value; *P < 0.05)

### Quantification of TNF-α and IL-1β in PICF

The data was normally distributed. The volumes of PICF were similar around the Zr and Ti healing abutments on day 0 but were significantly higher in tissues around the ZrL and TiL abutments than in those around the ZrN and TiN abutments on day 28 (Table 2).

**Table 2:** Comparison of PICF volumes around Zr and Ti healing abutments with or without ligation (mg)

		Day 0			Day 28			L			N		
		L	N	*P*	L	N	*P*	Day 0	Day 28	*P*	Day 0	Day 28	*P*
PICF	Zr	1.45 ± 0.32	1.45 ± 0.32	1.00	2.39 ± 0.51	1.12 ± 0.22	0.002*	1.45 ± 0.32	2.39 ± 0.51	0.121	1.45 ± 0.32	1.12 ± 0.22	0.133
	Ti	1.37 ± 0.16	1.58 ± 0.73	0.566	1.83 ± 0.29	1.08 ± 0.22	0.025*	1.37 ± 0.16	1.83 ± 0.29	0.220	1.58 ± 0.73	1.08 ± 0.22	0.416
	P	0.828	0.718		0.073	0.886		0.828	0.073		0.718	0.886	

Normal distribution test and two-way analysis of variance were applied

(*L* ligation, *N* non-ligation, *P* P value; *P < 0.05)

The levels of TNF-α and IL-1β in PICF around the Zr and Ti healing abutments, with or without ligation, are presented in Table 3. Although the TNF-α and IL-1β levels were similar around the Zr and Ti healing abutments on day 0, they were higher around the ZrL and TiL abutments than around the ZrN and TiN abutments on day 28. However, only the levels of TNF-α were significantly different between the ZrL and ZrN groups on day 28 (*P* = 0.022). The TNF-α level in PICF was significantly higher in TiL group on day 28 than that on day 0 (*P* = 0.011).

**Table 3:** TNF-α and IL-1β quantification in PICF around Zr and Ti healing abutments with or without ligation (pg/ml)

		Day 0			Day 28			L			N		
		L	N	*P*	L	N	*P*	Day 0	Day 28	*P*	Day 0	Day 28	*P*
TNF-α	Zr	4.25 ± 1.50	3.66 ± 1.40	0.550	7.85 ± 3.98	4.09 ± 1.64	0.022*	4.25 ± 1.50	7.85 ± 3.98	0.127	3.66 ± 1.40	4.09 ± 1.64	0.725
	Ti	3.32 ± 1.03	4.67 ± 2.36	0.468	6.95 ± 2.27	5.48 ± 1.95	0.343	3.32 ± 1.03	6.95 ± 2.27	0.011*	4.67 ± 2.36	5.48 ± 1.95	0.585
	P	0.535	0.482		0.556	0.369		0.535	0.556		0.482	0.369	
IL-1β	Zr	1.25 ± 0.22	1.32 ± 0.26	0.642	1.52 ± 0.28	1.12 ± 0.34	0.393	1.25 ± 0.22	1.52 ± 0.28	0.538	1.32 ± 0.26	1.12 ± 0.34	0.525
	Ti	1.11 ± 0.18	1.23 ± 0.16	0.426	1.67 ± 0.35	1.00 ± 0.20	0.173	1.11 ± 0.18	1.67 ± 0.35	0.396	1.23 ± 0.16	1.00 ± 0.20	0.429
	P	0.349	0.542		0.758	0.809		0.349	0.758		0.542	0.809	

Normal distribution test and two-way analysis of variance were applied

(*L* ligation, *N* non-ligation, *P* P value; *P < 0.05)

### Histological observations and histomorphometrical measurements

**Fig. 4:**
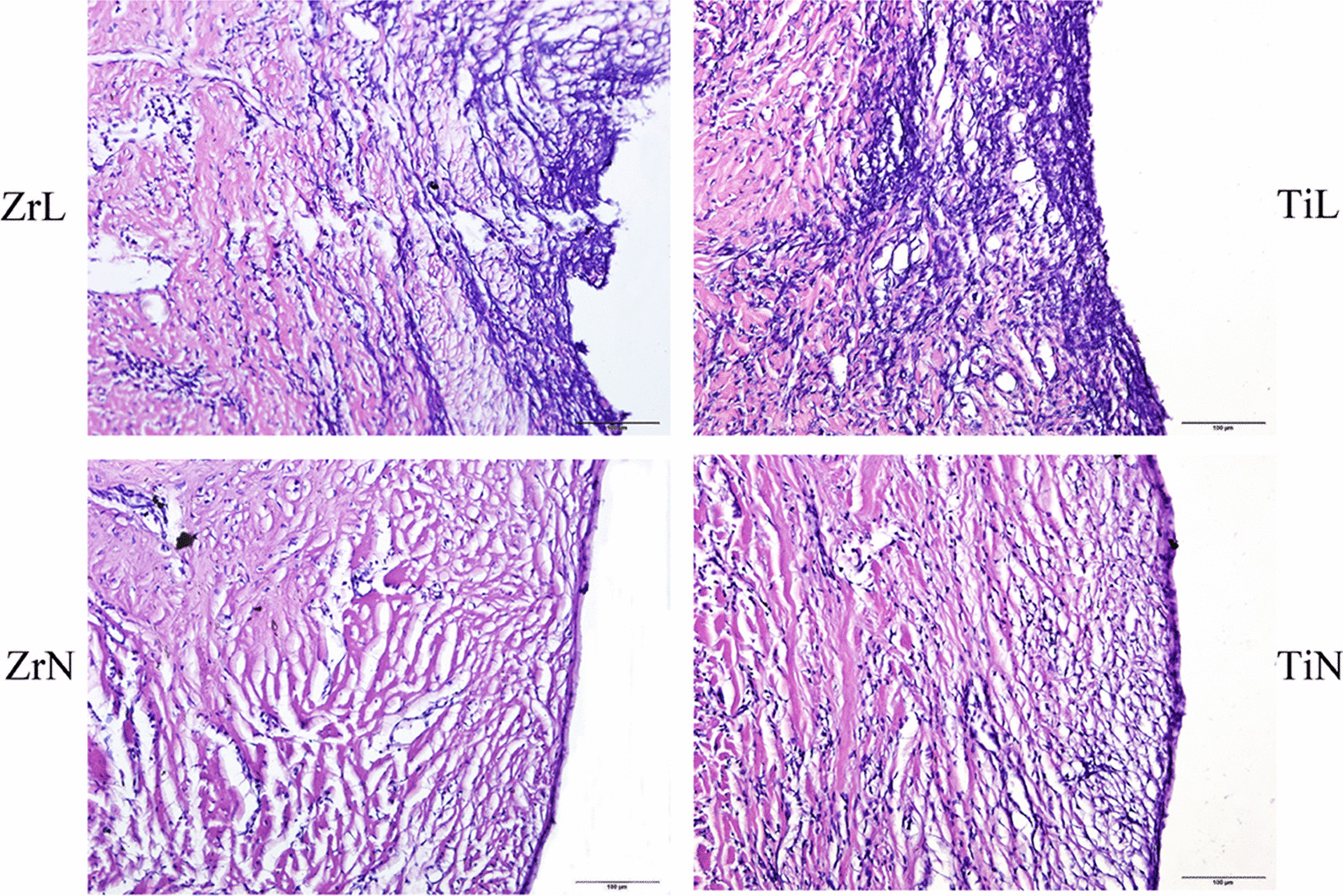
HE stain of soft tissues around Zr and Ti healing abutments with or without ligation (the bar = 100 µm) (ZrL: Zirconium healing abutments with ligation; ZrN: Zirconium healing abutments with non-ligation; TiL: Titanium healing abutments with ligation; TiN: Titanium healing abutments with non-ligation)

In the sections stained with HE, the collagen fiber bundles were oriented parallel to the surfaces of the ZrN and TiN healing abutments. The collagen fiber bundles were denser around the Zr healing abutments than around the Ti healing abutments, whereas fibroblasts were fewer around the Zr healing abutments than around the Ti healing abutments. Moreover, the epithelium proximate to the TiN healing abutments was more deeply stained than that proximate to the ZrN healing abutments. While the early soft tissue responses to the ZrN and TiN healing abutments were similar. The collagen fiber bundles adjacent to the ZrL and TiL healing abutments were disordered, and there was a higher number of fibroblasts stained deeper in the healing abutments with ligation than in those without ligation (Fig. 4).

**Fig. 5:**
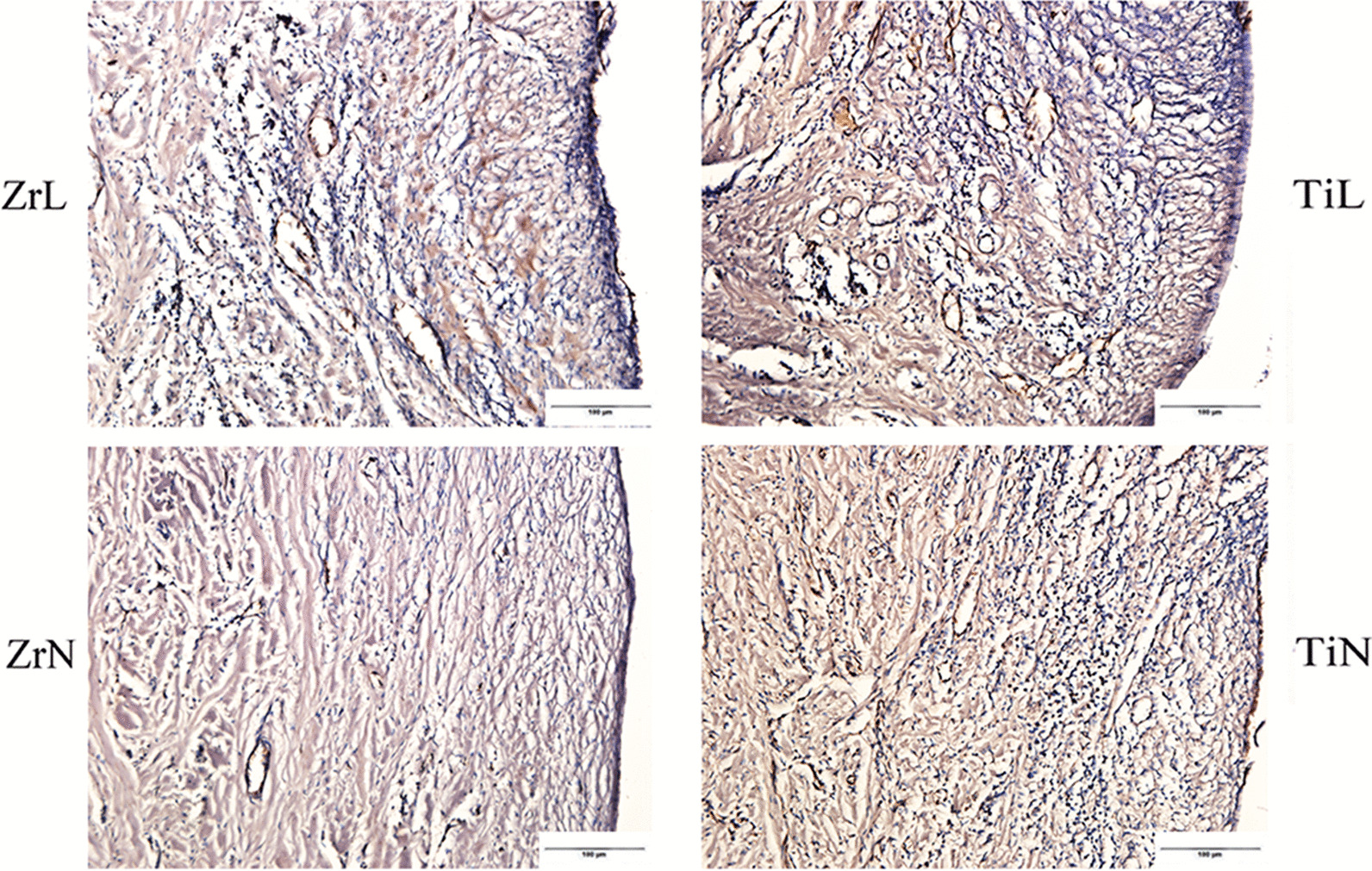
Immunohistochemical observation identifying TNF-α in soft tissues around the Zr and Ti healing abutments with or without ligation (the bar = 100 µm) (ZrL: Zirconium healing abutments with ligation; ZrN: Zirconium healing abutments with non-ligation; TiL: Titanium healing abutments with ligation; TiN: Titanium healing abutments with non-ligation)

**Fig. 6:**
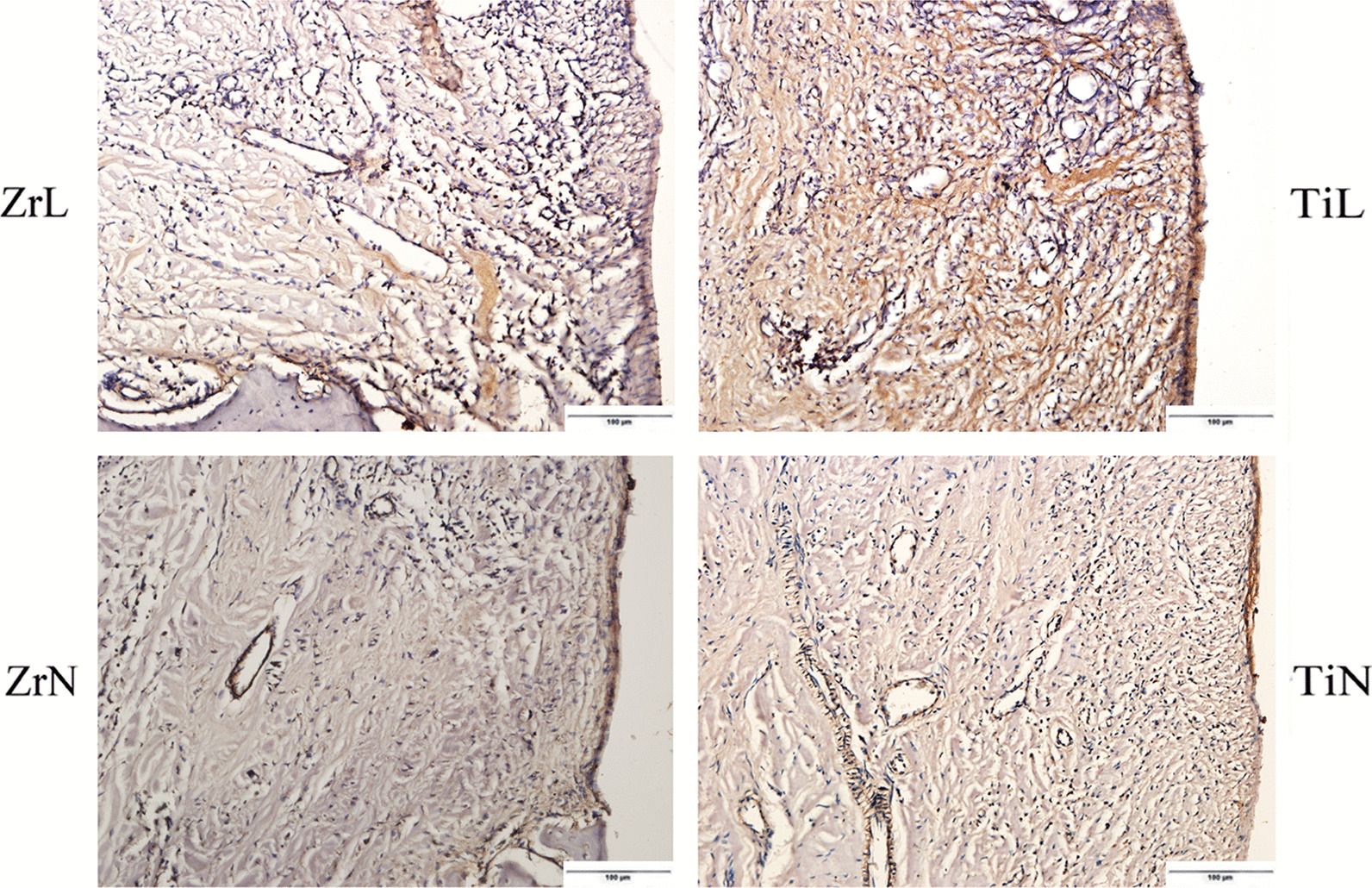
Immunohistochemical observation identifying IL-1β in soft tissues around Zr and Ti healing abutments with or without ligation (the bar = 100 µm) (ZrL: Zirconium healing abutments with ligation; ZrN: Zirconium healing abutments with non-ligation; TiL: Titanium healing abutments with ligation; TiN: Titanium healing abutments with non-ligation)

Immunohistochemical staining for TNF-α and IL-1β revealed the expression of positive inflammatory cells at the basement membrane zone, soft tissues adjacent to healing abutments, and endothelial cells of vessels in the vicinity (Figs. 5, 6). The inflammatory infiltrations were more obvious in tissues adjacent to the ZrL and TiL healing abutments than in those adjacent to the ZrN and TiN healing abutments. The mean amount of TNF-α positive inflammatory cells were 140.1 ± 25.4 and 160.1 ± 30.3 in the tissues adjacent to the ZrL and TiL healing abutments, respectively (*P* = 0.353), and 113.6 ± 11.2 and 114.8 ± 41.8 in those adjacent to the ZrN and TiN healing abutments, respectively (*P* = 0.963). Furthermore, the mean amounts of IL-1β positive inflammatory cells were 160.4 ± 45.1 and 214.9 ± 21.0 in the soft tissues adjacent to the ZrL and TiL healing abutments, respectively (*P* = 0.123), and 115.5 ± 23.1 and 134.6 ± 54.7 in those adjacent to the ZrN and TiN healing abutments, respectively (*P* = 0.563).

## Discussion

The properties of abutment materials can significantly influence the quality of mucosal attachment formation [2]. Recently, Zr abutments were introduced to improve the esthetics of implantation treatment of the maxillary anterior teeth, particularly in patients with thin mucosa [29]. However, there are only limited data on the soft tissue response to Zr, particularly in comparison with the response to Ti under peri-implantitis conditions. This within-subjects design study provides valid data comparing the expression of proinflammatory cytokines in PICF and the inflammatory infiltration of soft tissues around implant abutments fabricated from Zr and Ti with and without ligation. Importantly, we found no evidence of significant differences between the two types of biomaterials.

The results of this study demonstrates that peri-implant mucosal inflammation occurs around the ZrL and TiL healing abutments, and that soft tissues around these abutments becomes red and swollen. Furthermore, the GI was significantly higher in specimens with the ZrL and TiL healing abutments than in those with the ZrN and TiN healing abutments on day 28. However, no significant differences were found in PD, indicating that the type of inflammation induced by this procedure was peri-implant mucositis without bone loss. This result is consistent with that of a previous study in dogs, which indicated that peri-implant bone resorption occurred at 12 weeks after ligation [30].

We observed significantly increased volumes of PICF in the tissues around the ZrL and TiL healing abutments compared to those around the ZrN and TiN healing abutments on day 28, which was related to the occurrence of peri-implant mucositis. These results are in good accordance with previous studies, demonstrating a significantly increase in the volume of PICF after plaque accumulation [31, 32]. However, after oral hygiene behaviors were resumed, the volume decreased. These findings demonstrates that oral hygiene reduces peri-implant mucosal inflammation [31]. These data suggests that an increased volume of PICF could be a useful marker of the early inflammation in peri-implant soft tissues.

Investigations of the biochemical parameters in the gingival sulcus or PICF have become increasingly popular as this allows for monitoring of the health status of the gingiva and peri-implant mucosa [31, 32]. These biochemical methods allow for early diagnosis and potential applications in disease prevention. It has been shown that the levels of TNF-α and IL-1β in PICF were positively correlated associated with peri-implant mucositis and peri-implantitis [31, 33, 34]. TNF-α and IL-1β are primarily secreted by monocytes and macrophages, and are potent multifunctional cytokines acting as proinflammatory proteins in numerous signal transduction processes during inflammation. Therefore, we analyzed the levels of TNF-α and IL-1β in PICF in addition to clinical parameters.

In this study, we observed increased levels of TNF-α and IL-1β in the PICF of tissues around the ZrL and TiL healing abutments on day 28. However, only the levels of TNF-α were significantly different between the ZrL and ZrN groups on day 28, and the TNF-α level was significantly higher in TiL group on day 28 than that on day 0. Nevertheless, no significant differences in the levels of IL-1β were observed in this study. The findings of TNF-α and IL-1β in PICF obtained in the present study are in good agreement with the clinical findings, indicating that TNF-α could be useful markers for assessing early peri-implant health status with inflammation.

In this study, no differences were observed in the soft tissue inflammatory infiltration around the Zr and Ti healing abutments. However, fewer inflammatory cells were observed around the Zr healing abutments than around the Ti healing abutments. Similar results were also observed in a recent study conducted by Brakel et al. [20], who reported no differences in the inflammation grading scale score in peri-implant mucosa adjacent to the Zr and Ti abutments. Consistently, previous study in canines showed that peri-implant soft tissue histomorphology compositions were similar in implant abutments made of Zr and Ti after nine months of healing [35]. In addition, one recent human histology pilot study compared inflammatory responses of different dental implant abutment materials, and the results indicated that inflammation around Zr and Ti abutments were similar [36]. On the contrary, a study in canines conducted by Welander et al. [1] reported less inflammatory infiltration in the epithelium of the peri-implant mucosa around the Zr abutments compared to that around the Ti abutments. In a human histological study, Degidi et al. [18] reported significant elevations in the proinflammatory infiltrates (lymphocytes, plasma cells, and histiocytes) as well as an increased expression of vascular endothelial growth factor and nitric oxide synthase isoforms 1 and 3 in the tissues adjacent to the Ti healing abutments compared to those around the Zr healing abutments after a 6-month healing phase. Whether this observation was attributable to the favorable attachment properties of the surrounding connective tissues and the epithelium was not conclusively established. This is because the reduction in the inflammatory reactions may not merely be an expression of better insulation through the soft tissue, but may also be related to the well-established reduction in accumulation of bacteria found on ceramic surfaces [16, 17].

The immunohistochemistry assays in our study revealed the presence of inflammatory cells at the basement membrane zone, in the soft tissues adjacent to healing abutments, and in the small endothelial cells of vessels in the vicinity. These inflammatory cells may be related to Langerhans cells in the basal layer and vascular endothelial cells. Langerhans cells are lymphocyte antigen-presenting cells that play an important role in the early immune response of periodontitis or gingivitis [37], while vascular endothelial cells are involved in inflammatory processes via the release of proinflammatory cytokines.

## Conclusions

Within the limitations of this study, our findings indicate that soft tissue responses to Zr healing abutments with peri-implant mucositis are comparable to those to Ti healing abutments in vivo, providing a theoretical foundation for the clinical application of Zr.

## Data Availability

The data of this study are available from the corresponding author, HX, upon reasonable request.

## Abbreviations

EDTA: Ethylenediaminetetraacetic acid
ELISA: Enzyme-linked immunosorbent assay
GI: Gingival index
HE: Hematoxylin and eosin
L: Ligation
N: Non-ligation
PBS: Phosphate-buffered saline
PD: Probing depth
PI: Plaque index
PICF: Peri-implant crevicular fluid
SD: Standard deviation
Ti: Titanium
TiL: Ti healing abutments with ligation
TiN: Ti healing abutments without ligation
Zr: Zirconium oxide
ZrL: Zirconium healing abutments with ligation
ZrN: Zirconium healing abutments with non-ligation
